# Editorial to the Special Issue: “Synthesis of Organic Ligands and Their Metal Complexes in Medicinal Chemistry”

**DOI:** 10.3390/molecules27113644

**Published:** 2022-06-06

**Authors:** Irena Kostova

**Affiliations:** Department of Chemistry, Faculty of Pharmacy, Medical University, 2 Dunav St., 1000 Sofia, Bulgaria; irenakostova@yahoo.com

The field of medicinal (organic, bioinorganic, and coordination) chemistry as well as the interdisciplinary studies related to medicine is a rapidly developing area of study. In recent decades, numerous complexes containing metal ions and biologically active organic ligands have been reported, displaying the connection between organic and inorganic chemistry. Metal-based compounds possess many significant properties with various practical applications in many aspects of human life.

Considerable attention has been paid to designing new coordination complexes with improved pharmacological properties and a larger spectrum of activity. The application of new approaches such as theoretical methodologies broadly used in drug discovery is beneficial for the development of biologically active organic ligands and their metal-based complexes, which might potentially be used in clinical practice.

This Special Issue of *Molecules*, titled “Synthesis of Organic Ligands and Their Metal Complexes in Medicinal Chemistry”, is devoted to the following research topics within medicinal chemistry, specifically biologically active compounds: (i) new synthetic methodologies and analysis; (ii) theoretical, analytical, and physicochemical characterization; (iii) natural compounds; (iv) drug design; (v) in silico investigations; (vi) pharmacological activity; (vii) structure–function relationships; and other topics related to metal-based drugs.

Consistent with the high standards of this journal, the selected topics, both review articles and research papers, cover a broad array of topics in current research on various aspects of metal coordination complexes and their applications and present up-to-date information and recent advances in this area of research.

In this Special Issue, some of the papers deal with structure-based investigations of compounds useful for the discovery of novel drugs. Doytchinova et al. [1] designed a fragment-based library containing aromatic moieties and linkers originating from a set of novel acetylcholinesterase AChE inhibitors based on docking-based virtual screening. The authors used this library to design 1220 galantamine (GAL) derivatives following the model GAL-linker-aromatic fragment, screened them virtually for blood–brain barrier permeability, and docked them into rhAChE. Among the top 10 best-scored inhibitors, a representative lead molecule was selected and tested for anti-AChE activity and neurotoxicity. The in vitro tests for cytotoxicity and AChE inhibitory activity confirmed that the selected compound is a powerful non-toxic AChE inhibitor, 68 times more active than GAL, and could serve as a lead molecule for further optimization and development.

This Special Issue is mainly focused on recent efforts to prepare novel metal-based complexes and on reviews of some mechanistic insights into the pharmacological effects of these complexes, which are crucial to their medical success as well as to the rational design of new compounds with improved effectiveness. Rogalewicz et al. [2] reported the synthesis and physicochemical characterization of novel biologically active thiosemicarbazide derivative ligand L (N-[(phenylcarbamothioyl)amino]pyridine-3-carboxamide) and of the series of its Co(II), Ni(II), Cu(II), Zn(II), and Cd(II) complexes. The authors studied the pharmacokinetic profile of the compounds based on an ADMET analysis. The compounds exhibited potent biological activity against the A549 human cancer cell line. Barakat et al. [3] described the synthesis of a new quinazolinone-nitrate complex. The crystal structure of the complex was determined, and its structural and supramolecular structural aspects were analyzed. Using DFT calculations, the relative stability and intermolecular interactions of five suggested isomers were predicted. Biological screening of the antioxidant, anticancer, and antimicrobial activities of 4-hydroxyquinazoline and the corresponding quinazolinone-nitrate complex was discussed. Using the skeleton of biologically active orotic acid as a starting point to design potent structural analogues, new antioxidant metal complexes exhibiting antitumor properties were synthetized [4,5]. A new neodymium(III) complex of orotic acid was obtained, and its structure was determined by means of theoretical, analytical, and spectral analyses by Chiş et al. [4]. The cytotoxic effects of the compounds studied on different tumor cell lines were investigated. The participation of superoxide radical ion in cancer onset and progression is also well documented. Todorov et al. [5] synthetized new antioxidant lanthanum(III) and gallium(III) complexes of 5-aminoorotic acid. Their superoxide-scavenging behavior in the presence of a non-enzymatic source (potassium superoxide) was compared with that in the presence of an enzymatic source (X/XO). The enzymatic activity of XO was impacted by both complexes and the pure ligand in a concentration-dependent manner. In order to better relate the compounds’ chemical characteristics to XO inhibition, the compounds were docked in silico to XO, and it was proven that 5-aminoorotic acid and its complexes suppress superoxide production via XO inhibition.

The two review articles in this Special Issue presented an overview of the information available on two important topics [6,7]. The first is a comprehensive up-to-date review focused on natural compounds and their complexes, exhibiting a broad spectrum of pharmacological activities, and the second one deals with research on and the applications of platinum-based drugs.

The review by Budzisz et al. [6] highlighted the beneficial biological activities of plant compounds and their metal complexes, which are attributable to their different structural characteristics and substitution patterns. The first part of the review provides a comprehensive overview of exogenous and endogenous sources of reactive oxygen and nitrogen species, oxidative stress-mediated damage of lipids and proteins, and the protective roles of antioxidant defense systems, including plant-derived antioxidants. The strength of free radical inhibition has been discussed as being dependent on the chemical structure and substitution scheme, as well as on the functional groups that affect the possibility of binding with metal ions. Additionally, this review covers the anti-inflammatory and antimicrobial activities of flavonoids, chromones, coumarins, and their metal complexes, which support their applications in medicine, pharmacy, and cosmetology.

In recent decades, extensive research has been performed on platinum complexes. It is well known that platinum coordination compounds are among the most widely used anticancer chemotherapeutics. Although they have been extensively studied, a precise understanding of their mechanisms of action is still lacking. Moreover, the increasing resistance to existing drugs and their toxicity remain global problems in anticancer therapy. Consequently, the design of new antineoplastic drugs is a major challenge. Among the numerous cisplatin derivatives tested as antitumor agents, most of them have been rejected due to their toxicity. The comprehensive up-to-date review by Tsvetkova et al. [7] focused on the comparison of therapeutic combinations of the currently applied in clinical practice: cisplatin, carboplatin, oxaliplatin, nedaplatin, lobaplatin, heptaplatin, and satraplatin. It has been concluded that strategies for the development of platinum anticancer agents and for bypassing resistance to cisplatin derivatives and their toxicity include combination therapy, using Pt(IV) prodrugs, targeted nanocarriers, as well as a synergistic combination of the following: cisplatin derivatives with anticancer agents similar to fluorouracil, gemcitabine, cytarabine, fludarabine, pemetrexed, ifosfamide, irinotecan, topotecan, etoposide, amrubicin, doxorubicin, epirubicin, vinorelbine, docetaxel, paclitaxel, and nab-paclitaxel; modulators of resistant mechanisms; signaling protein inhibitors such as erlotinib, bortezomib, and everolimus; and immunotherapeutic drugs such as atezolizumab, avelumab, bevacizumab, cemiplimab, cetuximab, durvalumab, erlotinib, imatinib, necitumumab, nimotuzumab, nivolumab, onartuzumab, panitumumab, pembrolizumab, rilotumumab, trastuzumab, tremelimumab, and sintilimab.

Principally, the Special Issue “Synthesis of Organic Ligands and Their Metal Complexes in Medicinal Chemistry” provides a current perspective of the interdisciplinary and complex characteristics of this rapidly developing research area, as evident in the wide variety of problems and scientific approaches addressed. Considering the challenges in this exciting field of medicinal chemistry and drug discovery, this issue not only complements our knowledge on bioactive compounds but also may afford some novel ideas and motivation for further investigation of various prospective biologically active compounds having an impact on medical practice.
